# Evaluating Static Bone Cysts (SBCs) Through Long-Term Volumetric Analysis Using CBCT: A Study with 6-Month Follow-Up

**DOI:** 10.4317/jced.61099

**Published:** 2024-02-01

**Authors:** Hemant Sawhney, Manisha Singh, Ratna Banerjee, Geetanjali Gupta, Ashok Kumar, Jayant-Kumar Gahlot, Deepak Bhargava

**Affiliations:** 1 MDS. PhD Scholar in Center for Artificial Intelligence in Medicine, Imaging & Forensics, Sharda University. Associate Professor in Department of Oral Medicine, Diagnosis & Radiology. School of Dental Sciences, Sharda University, Greater Noida, U.P. – 201310; 2 MDS. Assistant Professor. Department of Oral Medicine, Diagnosis & Radiology. School of Dental Sciences, Sharda University, Greater Noida, U.P. – 201310; 3 Associate Professor. Department of Decision Sciences, Sharda School of Business Studies, Sharda University, Greater Noida, U.P – 201310; 4 Post Graduate Student. Department of Periodontology. Kalka Dental College, Meerut, U.P; 5 Head, Center for Artificial Intelligence in Medicine, Imaging & Forensics. Professor, Dept of Physics, School of Basic Sciences & Research (SBSR). Sharda University, Greater Noida, U.P. – 201310; 6 Assistant Professor. Department of Oral Medicine, Diagnosis & Radiology. School of Dental Sciences, Sharda University, Greater Noida, U.P. – 201310; 7 Head of Department. Department of Oral Pathology & Microbiology. School of Dental Sciences, Sharda University, Greater Noida, U.P. – 201310

## Abstract

**Background:**

Salivary gland bone defects are static lesions which are rare entities, generally asymptomatic and found in routine imaging exams. However, in atypical cases or when misdiagnosed, surgical intervention is carried out. Purpose: a) The study is intended to investigate the frequency of SBC’s and to describe the radiological characteristics of its subtypes; b) To evaluate the efficacy of volumetric analysis tool in CBCT and; c) To describe the confirmative role of CBCT in the diagnosis of SBC’s without the need for surgical intervention.

**Material and Methods:**

The study was conducted on 11 subjects with SBC detected on 3304 panoramic radiographs. CBCT images for each patient were captured at baseline and at an interval of 6 months. Radiographic sub-types of SBC based on the relationship to mandibular canal and bucco-lingual expansion were studied. Files in DICOM format were transferred into OnDemand 3D program (Cybermed Co, Seoul, Korea) and volumes in mm3 of the cavities were measured by 2 observers at both intervals. Inter and intra reliability of volumetric measurements between observers was compared using correlation coefficient and student t test.

**Results:**

There were 8 males and 3 females who had SBC in this study (age range: 22-70 years). According to the relationship of SBC with mandibular canal, most SBCs were Type 1 (63.5 %) followed by Type 2 & 3 (18.5 %) each. The total volume of SBC in patients ranged from 146 mm3 to 650 mm3 (mean: 332.5 mm3). There was no significant difference between observers for volume measurements at baseline and at 6 months interval (*p*>0.05).

**Conclusions:**

Based on the results of this study, CBCT should be considered as a definitive diagnostic modality for volumetric analysis of SBCs. Over diagnosis, unnecessary surgical exploration and agony to patients can be avoided using this tool.

** Key words:**Stafne bone cavity, CBCT, Volumetric analysis, salivary gland, panoramic radiography.

## Introduction

Mandibular radiolucencies are present in a variety of situations. They fall into developmental, metabolic, traumatic, infectious, and neoplastic categories. Differential diagnosis of these “lesions” is arrived at through signs, symptoms, history, radiographic presentation, and other diagnostic tools. Stafne’s bone cyst or Stafne bone defect is a rare mandibular bone defect that presents as an asymptomatic unilateral radiolucent cavity in the posterior region of the mandible below the inferior alveolar canal (1) and was first reported by Stafne in 1942. The other names of Stafne’s bone cyst are static bone cyst, lingual mandibular bone defect, idiopathic bone cavity, and lingual mandibular bone depression. It commonly presents as a well-demarcated, asymptomatic, unilateral radiolucency that indicates lingual invagination of the cortical bone (2).

The prevalence of Stafne’s bone cyst varies from 0.10% to 0.48% and is more common in males (3). The shape of the Stafne’s bone cyst varies from round to oval and the size of the lesion is about 1-3 cm (4). Four topographical variants of Stafne’s cyst are found with the most common variety being the posterior variant, and these lesions are present between mandibular angle and third molar (5). Anterior variant is less common when compared to the posterior variant located between the lower incisor and premolar areas. The third and fourth variants are in the buccal and lingual aspect of ascending ramus of the mandible. The most accepted theory about Stafne’s cyst is that these cavities develop because of localized pressure induced atrophy of the lingual surface of the mandible from the adjacent salivary gland (6). Stafne’s cyst usually contains ectopic salivary gland tissue and does not require surgery (7).

Stafne cysts are detected radiographically during routine dental radiographs. Stafne’s cysts are more commonly diagnosed by Orthopantomography. On orthopantomography, it is not possible to differentiate the involvement of buccal or lingual cortices by the Stafne cavities. Advanced radiology like Cone Beam Computed Tomography (CBCT) has the advantage of identifying the peripheral origin of the lesion and the relationship of the lesion with both buccal and lingual cortex and can help in differentiating Stafne’s cyst from other cysts and tumours (8). CBCT scans produce three-dimensional images with low radiation doses and high contrast to identify SBCs. Moreover, advanced tools are being incorporated in the imaging software which easily help the operator to segment, visualize and assess these defects volumetrically.

SBCs can sometimes result in a diagnostic dilemma. The differential diagnosis of Stafne’s cyst includes odontogenic cyst, fibrous dysplasia, brown tumor of hyperparathyroidism, ameloblastoma, basal cell nevus syndrome, and giant cell tumor (9) Volumetric measurements using CBCT proves to be a useful tool in evaluating the volumetric size of the various oral lesions including periapical abscess, cysts, and tumors (10). The detection of the volume of the lesion is important when the dimensions of lesions are compared with the help of follow-up radiographs (11).

This paper presents rare cases of Stafne’s bone defects which are diagnosed on a routine panoramic dental radiograph carried out for different dental treatments. This article will discuss the different variants of the Stafne Bone Cavity and the importance of CBCT in providing a definitive diagnosis of SBC patients and how these patients are deferred from surgery.

-Objectives

In the present study, the main objectives are to investigate the frequency of Stafne Bone Cyst and describe the radiological characteristics of its subtypes, evaluate the efficacy of the volumetric analysis tool in CBCT, and describe the confirmative role of CBCT in the diagnosis of SBCs without the need for surgical intervention.

## Material and Methods

A total of 3304 digital panoramic radiographs with an age group of 22-70 years fulfilling the inclusion and exclusion criteria were screened for the presence of SBCs. The digital panoramic radiographs were evaluated for 1 year span ranging from May 2022 to May 2023 for the presence of SBCs. The radiographs screened were done either for clinical or diagnostic purposes at the Department of Oral Medicine and Radiology. The radiographs were done by Carestream CS 9300 Digital Panoramic and Cephalometric machine with standard exposure parameters of 75 kVp, 12 mA, and 15.2s. All the radiographs were viewed in DICOM Image Viewer (CS 3D Suite (v3.10.12) on monitor (Dell Inspiron 15 7573, 4K, 3840x2160 pixel, 32bit color screen, UHD Touchscreen, Backlit, 2.4GHz Intel Duo Core, 4 GB RAM, Nvidia Graphic Card). Image enhancement tools were used to make the best possible diagnosis for the suspected lesions.

The inclusion criteria included high resolution images showing well demarcated sharply corticated round or oval radiolucency of the mandible with a typical location below the inferior alveolar nerve canal but is limited by the angle of the mandible. Repeat radiographs for the same patient in that duration (to avoid over-estimation) and radiographs having other cystic lesions / tumors / fractures near the angle and lower border of the mandible were excluded from the study.

Panoramic radiographs were examined for the presence of SBCs by two radiologists having more than 10 years of dental radiographic image interpretation experience. The entire screening protocol was blinded for the radiologists and was done twice with an interval of 1 week. Out of 3304 panoramic radiographs, 11 panoramic radiographs showed the presence of SBCs as an elliptical, oval, or homogeneous well-defined unilocular radiolucency with well-defined corticated borders located in the mandible below the inferior alveolar canal. SBCs were considered as the radiographic diagnosis based on location and shape (12). A small FOV low-dose CBCT was carried out for 11 subjects to confirm the location, shape, relationship with cortices and volume of SBCs (13) at baseline and after 6 months interval. The ethical clearance was obtained from the Institution’s Ethical board and research committee. The study fulfils the guidelines of the Declaration of Helsinki about Ethical Principles for Medical Research involving human subjects.

CBCT scans were performed at the Department of Oral Medicine and Radiology using Carestream CS 9300 Select 3D digital imaging system Dental Carestream LLC, Atlanta, GA, USA) at baseline and repeated at an interval of 6 months from baseline. Images of CBCT scan were obtained at 70-80 kV, 10-12 mA, FOV size of 5x5 to 8x8mm, and with a resolution of 150-180µm. The CT DICOM (Digital imaging and communication in medicine) images were transferred into 3D reconstructed images using the OnDemand 3D program (Cybermed Co, Seoul, Korea).

**Figure 1:**
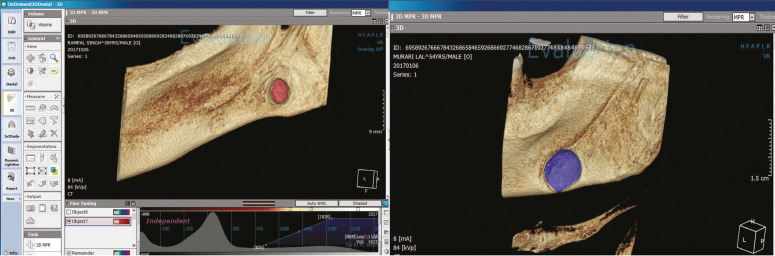
Volume measurement using segment tool on OnDemand 3D software.

The volume measurement on OnDemand 3D program involved selecting region of interest (ROI) by manually selecting the most exterior point of the concavities of the SBCs followed by segmenting it using Segment 3D tool. While using the segment tool, it was ensured that proper threshold levels were adjusted. 3D model was generated by using this tool and volume in mm3 was obtained with the help of the software, (Fig. 1). Volume calculation was done by the same two radiologists at the baseline and 6 months after the baseline. Both the radiologists have more than 4 years of experience in handling, viewing & interpreting CBCT images and in using segmentation software. The two radiologists were blinded again, and they separately calculated the volume in mm3 in the cavities at both instances. The volume in mm3 was measured twice at an interval of 1 week to eliminate the intra-observer bias.

This study evaluated the relationship of the mandibular canal with SBCs based on the three radiological types by Reuter I, 1998 (14).

• Type 1 - Mandibular canal is separated from the SBC and it is not reaching the buccal cortex.

• Type 2 - Mandibular canal is in contact with SBC and it is reaching the buccal cortex without expansion.

• Type 3 - Mandibular canal goes through the SBC with expansion of the buccal corterx.

Cronbach’s Alpha tests were used to test intra-observer agreement. All data was subjected to the Student paired “t” test and Pearson correlation coefficient test using SPSS Software (Version 16.0) in Windows for the correlation of volume in the cavities at baseline and 6 month interval.

## Results

A total of 3304 panoramic radiographs were screened and from them 11 radiographs had SBCs, out of which 8 males (72%) and 3 females (28%) were present with the age range of 25-67 years. All 11 patients were noted with unilateral SBCs (100%), out of which 7 were seen on the left side (64%) and 4 were seen on the right side (36%). In the present study, all SBCs were noted with the posterior variant, between the mandibular angle and third molar (100%). Based on the relation between SBCs and the mandibular canal, 7 patients with SBCs showed Type 1 (63%), 2 patients showed Type 2 and Type 3 relation each (18.5%).

The volume in mm3 at baseline by Radiologist 1 and Radiologist 2 varies from 160 mm3 to 650 mm3 and 149 mm3 to 649 mm3 respectively (Table 1). The volume in mm3 after 6 months by Radiologist 1 and Radiologist 2 varies from 162 mm3 to 642 mm3 and 146 mm3 to 632 mm3 respectively (Table 2). Paired sample t-test showed no significant difference (*p*>0.05) in the volume in mm3 at baseline and after 6 months measured by Radiologists 1 & 2 (Table 3). Karl Pearson’s coefficient of correlation showed a high positive linear association between Radiologist 1 and Radiologist 2 in their measurements. Also, the correlation coefficient is significant (*p*>0.05) (Table 4).

**Table 1:**
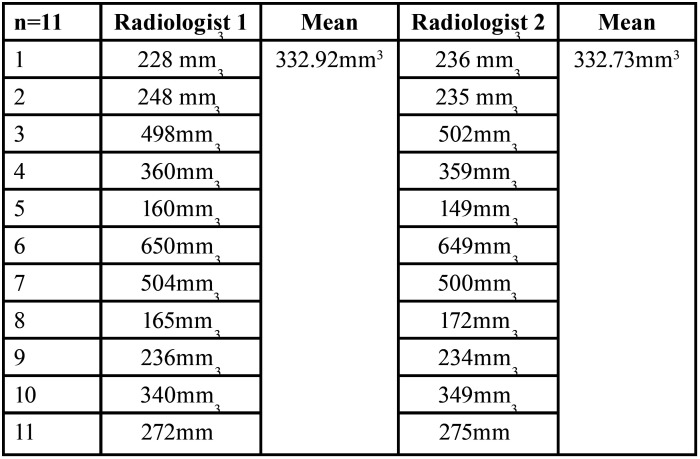
Volume assessment at baseline between Radiologist 1 and 2.

**Table 2:**
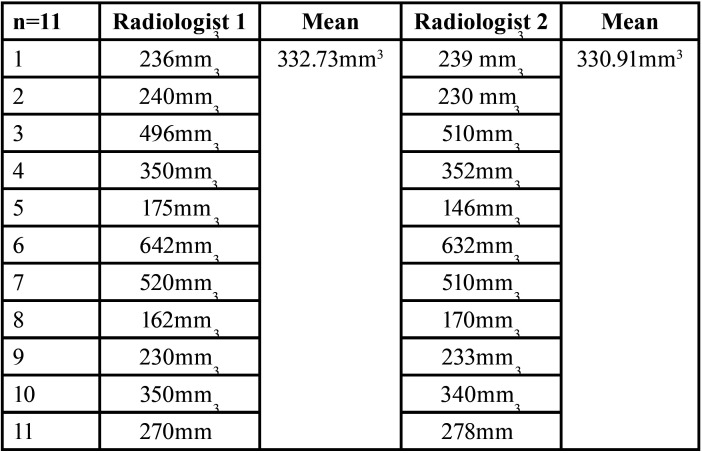
Volume assessment after 6 months between Radiologist 1 and 2.

**Table 3:**
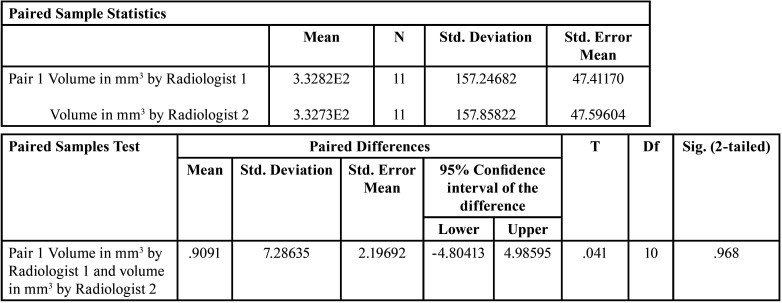
T Test.

**Table 4:**
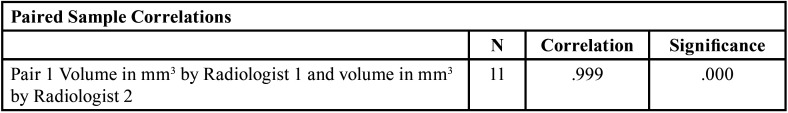
Karl Pearson’s coefficient of correlation (Moment correlation between Radiologist 1 and Radiologist 2).

## Discussion

The incidence of SBCs when detected on radiographs is generally low (0.1%-0.48%). (15) The prevalence of SBCs in our study is 0.3%. Koc A *et al*. in their study revealed SBC prevalence of 0.58%, with an anterior variant prevalence of 0.04%, and a posterior variant prevalence being 0.54%. (16) A total of 11 SBC’s was diagnosed on the 3304 panoramic radiographs and all being the posterior lingual variant in our study. Bilateral presentation was not noted in our study. Our study demonstrated the male: female ratio of 8:3 with 36% originating from the right mandible and 64% from the left mandible. Koc *et al*. in their study reported a male: female ratio of 13 : 1; 26.7% located on the left side and 73.3% on the right side. The findings are in accordance with our study that incidence of SBC’s is higher in males. However, our study results and other studies reveal that there is no site predilection for occurrence of SBCs which could be attributed to different ethnicities and geographical areas of the study (16). SBCs are pseudo cysts and are important to maxillofacial radiologists for the correct diagnosis and in ruling out other pathological cysts and tumors. The diagnosis of SBCs is often confused with odontogenic cysts, fibrous dysplasia, brown tumor of hyperparathyroidism, ameloblastoma, basal cell nevus syndrome, and giant cell tumor (17). It has been observed that SBCs are accidental finding on a routine panoramic radiograph. Panoramic radiography can only describe the position and extent of the SBCs, however, the accurate size, involvement of mandibular canal and the cortical bone can only be defined by three-dimensional images of Computed Tomography. CT imaging of SBCs in certain case reports have shown that they contain salivary gland tissue and some reports showing that they contain only fat or soft tissue. However, CT imaging has lot of inherent disadvantages ranging from higher radiation dose, high cost and possible contrast agent reactions. MR imaging and sialography remains a valuable tool in confirmatory diagnosis of Stafne defects but are rarely being advised due to high cost, technical expertise, and patient discomfort (18). Cone beam computed tomography (CBCT) gives a detailed bony resolution of the concerned area, having a low cost and less radiation dose to the patient and is a non-invasive imaging modality (19).

**Figure 2:**
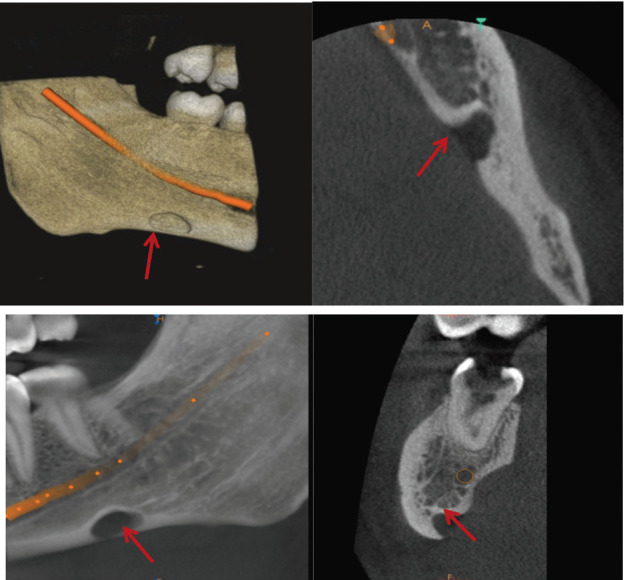
TYPE-1 SBC; Mandibular canal is separated from SBC.

**Figure 3:**
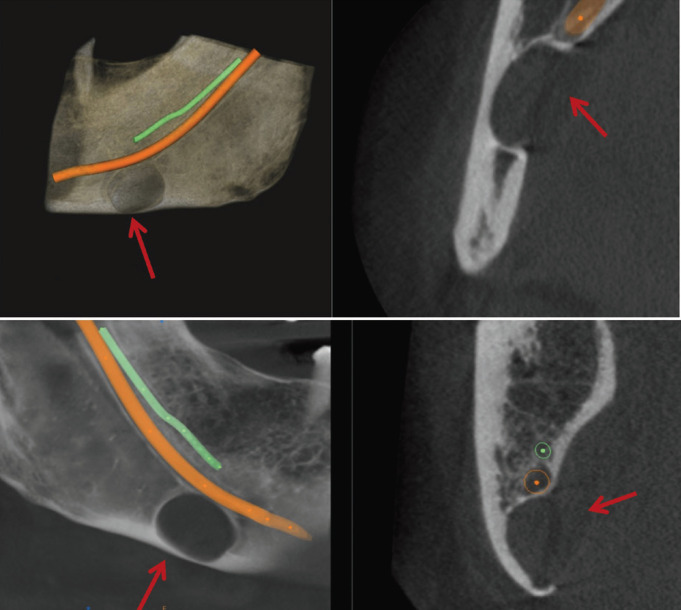
TYPE-2 SBC; Mandibular canal is in contact with SBC.

**Figure 4:**
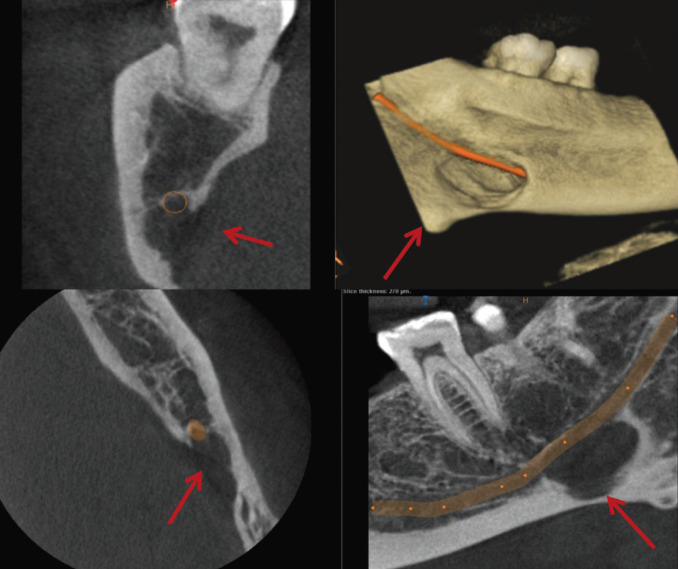
TYPE-3 SBC; Mandibular canal goes through the SBC.

The most common variant is the posterior variant, and this study showed all 11 patients with SBCs to be associated with this variant. Previous literature stated the age group associated with Stafne’s cyst was from 40-60 years. In this study, the age group varied from 25 years to 67 years. Oikarinen VJ *et al*. mentioned that 3 cases out of 10000 panoramic radiographs showed Type 1 SBC (20). Adisen MZ *et al*. in 2015 stated 2 cases of Type 2 SBC where the mandibular canal was in contact with SBC and 3 cases of Type 3 SBC where the mandibular canal goes through SBC. (21) Most of the SBCs in our study were of Type 1 (n=7) where the mandibular canal was separated from SBC followed by Type 2 and Type 3 (n=2 each), (Figs 2-4).

Based on the effects of these cavities on the cortical and trabecular bone, SBCs were classified into three types by Ariji *et al*. in 1993 (22). In Type 1, the bottom of the concavity did not reach the buccal cortical plate. In Type 2, the concavity reached the buccal cortical plate, but there was no cortical expansion or distortion. In Type 3, it was characterized by a buccal expansion of the cortical plate. The proposed modification of this classification was given in the year 2020 by Chaudhary A (23) where she pressed a need for bi-cortical perforation to be included as a modification. Our study saw SBCs of all the three radiological variants.

The second objective of our study was to evaluate the efficacy of volume measuring tool in CBCT and to determine if such a modality can be utilized as a follow up tool for such defects where radiological diagnosis must be considered sufficient avoiding surgical exploration. Several authors have compared the accuracy of different third-party software for measurement of airway volume of oropharynx, lesions of bone, periapical lesions and simulated bone lesions on bovine bones (24,25). Several PC programs like Mimics, Dolphin3D, ITK-Snap, OsiriX, Ondemand 3D, Invivo, etc. have proved to be equally reliable. Adisen MZ *et al*. in 2015 evaluated the volumetric measurements of 14 SBCs on CBCT by 2 observers at 1 month interval (21). They concluded that there was no significant difference between the two observers in volume measurement. The total volume of SBCs in their study ranged from 160mm3 to 520mm3 with a mean of 361.7mm3. In the current study CBCT data imported in OnDemand 3D software was used as a measuring tool for the volumetric size measurements of SBCs at the baseline and after 6 months by 2 radiologists. The mean volume measured by radiologist 1 at baseline was 332.82mm3 and at 6-month interval was 333.73mm3. Volume measured by radiologist 2 at baseline and at 6-month interval was 332.73mm3 and 330.91mm3 respectively. There was no significant difference between the volumetric size in mm3 at the baseline and after 6 months (*p*>0.05).

## Conclusions

Oral medicine and radiology specialists should be aware of such salivary gland defects presenting as radiolucent lesions present in the posterior region of the mandible. Panoramic radiographs can diagnose the presence of SBCs as an incidental finding but for the definitive diagnosis, three-dimensional cone beam imaging is required. CBCT tool for measuring volume at biannual intervals for any static lesions in the jaw will help in preventing unwarranted surgical treatment and will reduce the agony to the patients. Our follow-up study of asymptomatic SBC’s patients and three dimensions images of CBCT has proven to be beneficial in the diagnosis of SBCs. Further studies should be done to correlate the clinical findings with the radiological diagnosis of SBCs patients. Some SBC patients may be symptomatic and have positive clinical findings that would require surgical treatment with histopathological examination.
